# Evaluation of using a graphic novel *Vivian* in nursing curriculum from the perspectives of nurse educators: a three-country qualitative study

**DOI:** 10.1186/s12904-025-01907-y

**Published:** 2025-10-24

**Authors:** Ashwini Deshmukh, Alison Gayton, Lisa Williams, Carina Werkander Harstäde, Jane Nicol, Tatiana Tavares, Susan Waterworth, Natalie Anderson, Merryn Gott, Ping Guo

**Affiliations:** 1 Department of Nursing and Midwifery, School of Health Sciences, University of Birmingham, Birmingham, UK; 2 University of Auckland, Auckland, New Zealand; 3 Linnaeus University Växjö, Växjö, Sweden; 4 St Richard’s Hospice, Worcester, UK; 5 Auckland University of Technology, Auckland, New Zealand

**Keywords:** Palliative care, Graphic novel, Undergraduate nurse education, Qualitative study, Graphic medicine

## Abstract

**Background:**

Graphic novels - full-length stories published in comic-strip format are a proven method for putting student nurses ‘into the shoes’ of health service users and offer nurse educators an alternative to traditional modes of instruction. Their use can support the teaching of palliative care related topics, which remains a challenge in nurse education. This study aimed to explore the views and perspectives of nurse educators about how the graphic novel *Vivian* could be used to prepare undergraduate nursing students for palliative care and beyond.

**Methods:**

Focus groups and individual interviews were conducted with 12 nurse educators at universities in the United Kingdom, New Zealand and Sweden and analysed using thematic analysis.

**Results:**

Four themes were generated: (1) impact of visual storytelling, (2) arts-based learning in palliative care, (3) refinement of the graphic novel, (4) recommendations about future graphic novels. Participants noted *Vivian*’s usefulness for teaching issues related to palliative care and the health care system, including gender inequities, ageing, and economic crises.

**Conclusions:**

There is scope to use arts-based learning when engaging audiences from diverse backgrounds. The educators stressed *Vivian* could help students think ‘outside of the box’ and stressed the importance of a teaching and learning approach that extended beyond textbooks to include other interactive forms. Future research is needed to adapt and refine the graphic novel by considering social and cultural contexts and evaluate how the graphic novel is implemented in nurse education across different settings and countries.

**Supplementary Information:**

The online version contains supplementary material available at 10.1186/s12904-025-01907-y.

## Background

Nurse educators play an important role in preparing undergraduate nursing students for roles in palliative care. However, they often face challenges of teaching about palliative care, particularly sensitive and complex concepts such as equity, person-centred care, and dignity in grief and bereavement [1]. To build and achieve the competences required in supporting patients towards the end of life and their family, it is imperative that these essential concepts are included in the nursing curriculum [2]. The lack of both didactic education and clinical experiences of palliative and end-of-life care has made nursing students unprepared when they cared for a patient who was dying [3]. Despite the increasing recognition of the importance of nurse education in palliative care, there is limited knowledge on how to effectively teach undergraduate nursing students in this area. Educators are still exploring innovative ways to facilitate students learning how to provide person-centred high quality palliative care.

Evidence across the disciplines shows that activities involving graphic novels could potentially stimulate engagement and enjoyment in learning, help students to memorise and clarify key concepts, and enhance critical thinking [4]. The last few years have seen an emergence of graphic novels - full-length stories published in comic-strip format, which have been used as teaching and learning tools, to develop professionalism and professional values in, for instance, medical and nurse education [5]. Graphic novels use images with enhanced print and other forms of visual information giving a new perspective to storytelling [6]. This allows the reader to relate to the story, pictures, plot and style of presentation. They convey meaning with three types of symbols - linguistic, spatial and visual: the linguistic symbol helps with letters, words, texts; the visual symbol uses images and pictures, and the spatial symbol employs position, direction, and layout in the story [7]. Graphic novels take the reader across time and space and help the reader to use their imagination [7, 8], which provides an additional medium of communicating important concepts [9].

Little is known about the use of graphic novels in nurse education. However, a scoping review identified 29 articles examining how graphic novels and comics are used in medical training, particularly in some areas of clinical medicine (e.g., palliative care, difficult communication, and rare diseases) [10]. The findings showed that graphic novels and comics proved to be a possible way to provide a vicarious experience for learning, and can support the achievement of cognitive outcomes, as well as soft skills and professionalism. Graphic novels not only work as a tool for students to improve their skills, observation, analysis, reflection, critical thinking and thinking out of the box [11], but they also enable students to look at the disease and illness, and the experiences associated with the illnesses, from different perspectives [12, 13].

**Fig. 1:**
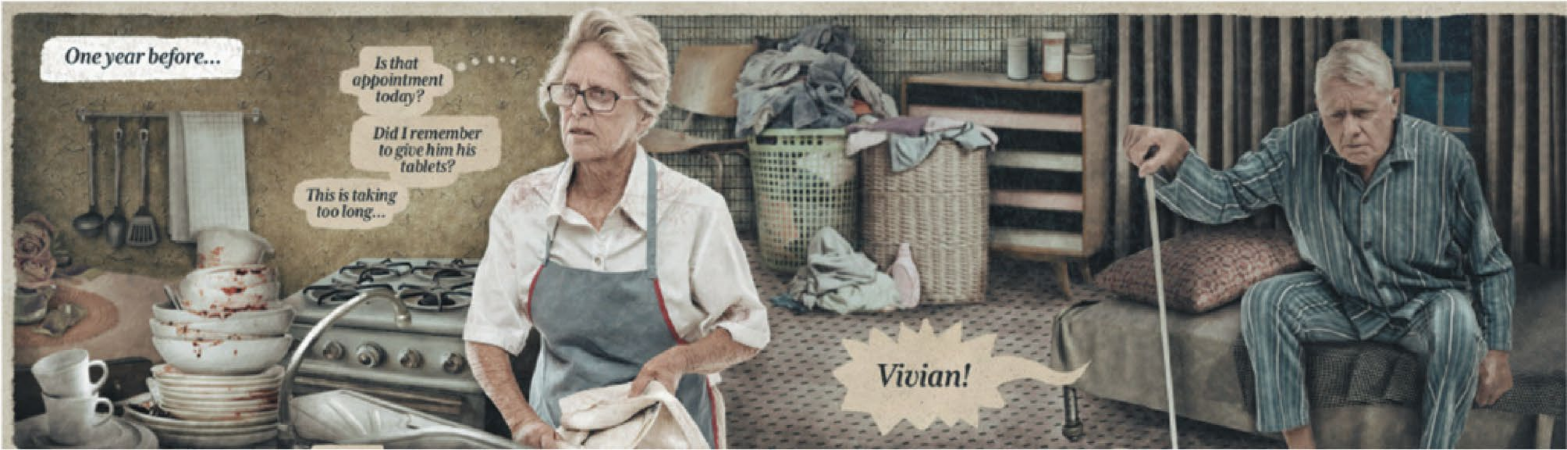
Illustration of a scenario from the graphic novel – *Vivian*

Topics such as palliative care extend beyond textbook materials [8]. *Vivian* was developed to teach undergraduate nursing students about palliative care as a teaching aid. The novel tells the story of Vivian, a white older woman who cared for her husband during his last stage of illness and faced her own health issues (Fig. 1). The story illustrates social, financial, and other constraints Vivian faces and the lack of information and support provided by healthcare professionals. Scenarios portrayed within the graphic novel shed light on topics such as gender disparities in palliative care treatment, ageism and poverty. The visual style employed in *Vivian* relies on photomontage to present and subtly alter the realistic portrayal of everyday life. The use of thought bubbles and bubbles with things people say give the graphic novel insights into not only conversation but Vivian’s thoughts and feelings. *Vivian* is freely available to download in PDF form via: https://www.tearairesearchgroup.net/newsarchive/introducing-vivian?rq=vivian.

The graphic novel – *Vivian* has been evaluated among undergraduate nursing students in three countries - New Zealand, Sweden and England. The findings have been published in a separate paper, suggesting various positive impacts on student learning experiences and outcomes [14]. As a nurse educator it is important to understand that graphic novels could be an effective and efficient tool for teaching such topics and use of these tools could improve understanding of students [10, 14]. Therefore, this study aimed to explore the views and perspectives of nurse educators about how the graphic novel - *Vivian* could be used to teach undergraduate nursing students about palliative care and beyond.

## Methods

### Study design

A multinational, qualitative study was conducted using a combination of semi-structured interviews and focus groups. The consolidated criteria for reporting qualitative research guidelines (COREQ) was followed [15]. A constructivist paradigm was used which identifies relativist ontology and subjective epistemology [16]. The same reality is experienced and expressed differently by people owing to their individual role in society and their cultural and ethnic background. Similarly, constructivism helps in building knowledge shared within the community and by its people; the interactions and results based on their experiences [17].

### Methodological rigour

The project team members from three countries met on a regular basis to monitor and review the detailed progress of the study and make recommendations for the overall direction of the project, strategies of data collection/analysis and beyond. In establishing trustworthiness, Lincoln and Guba’s four-dimension criteria (credibility, dependability, confirmability and transferability) were adapted to assess and ensure the rigour of this multi-country qualitative research [18–20]. We carefully planned and implemented strategies such as defining adequate sampling approach; obtaining inclusive participation; preparing for data collection and analysis methods; reaching information power; training and supporting early career researchers; and ensuring high levels of consistency and inter-coder agreement.

### Participants and setting

Purposive sampling was used to recruit nurse educators with diverse characteristics (e.g., age, gender, background) from the University of Birmingham in the United Kingdom, The University of Auckland in New Zealand, and Linnaeus University in Sweden. All the nurse educators in the three participating universities were invited if they had clinical and/or teaching experience of palliative care. Purposive sampling helped researchers to explore different perspectives to generate new knowledge based on the data [21]. Recruitment of participants took place between March 2023 - January 2024.

### Data collection

Five semi-structured interviews and two focus groups (face to face or online) were conducted by the core research team (PG, AD, LW, SW, CHW) across three sites. We adopted a pragmatic approach to maximising participation opportunities whereby multiple ways were provided to participate, including in-person, online, individual interviews, and focus groups to cater to different needs and preferences of our participants. In addition, interviews delved deep into personal views and experiences of nurse educators, while focus groups provided complementary data to reveal how perspectives were shared and debated within a group. Combining both the individual in-depth interviews and dynamic group discussion allowed us to gather a richer and more comprehensive understanding of the topic and enabled us to explore both shared perspectives and nuanced individual experiences simultaneously [22].

An interview guide was developed by the research group and used at all sites to maintain consistency of the data (supplementary file 1). A participant information sheet and consent form were sent electronically to all participants prior to their interview and written informed consent was obtained from all the participants before the interview or focus group. Details about data collection at each site are summarised below.

At the University of Birmingham, United Kingdom, email invitations about the study were sent to the nurse educators stating details about the study. Potential participants who were willing to join the study, signed consent form and were sent a PDF copy of *Vivian* electronically via email to read before the focus group discussion. The focus group was conducted online via zoom by AD and PG and digitally audio recorded and transcribed verbatim by AD. Transcription was checked by PG & AG for accuracy of the data. Confidentiality was maintained throughout this process by giving ID numbers to the participants instead of using the names.

At the Linnaeus University, Sweden, the nurse educators in the palliative course were approached and invited to participate in a focus group discussion. The focus group discussion was conducted and recorded with consent online via zoom in Swedish language and translated and transcribed verbatim in English by CWH.

At the University of Auckland, New Zealand, study invitations were sent via email and participants were approached face-to-face by LW asking them if they would be willing to participate in this study. After written informed consent was obtained, individual interviews were conducted by LW. Transcription was completed by a professional transcriber who signed a confidentiality agreement.

Data collection continued until it was collaboratively decided by PG and each on-site research team that sufficient information power was reached [23]. The interviewers kept written reflections to not only record contextual factors and emergent themes, but they were also encouraged to reflect on how their characteristics, experiences, and communication skills may have potentially affected the data collection. These reflections were later discussed in team meetings and considered when each transcript was coded and at later stages of the analysis process.

### Data analysis

Data were initially coded and analysed by an early career researcher (AD) and cross checked by senior team members (AG and PG). The three researchers (AD, AG, PG) independently examined one full transcript to develop a coding framework and then AD applied the coding framework to analyse the remaining transcripts. Reflexive thematic analysis approach was used [24] and data analysis was performed using the software NVivo (version 10). Data were grouped and compared enabling generation of common themes and sub-themes [25, 26]. The generated themes (including overlapping themes merged and themes containing few quotations) were discussed and agreed with all the team members to improve the dependability and confirmability of the study.

### Ethical considerations

Relevant ethics approvals were received from the University of Birmingham Research Ethics Committee (ERN_20-1884), the Ethical Advisory Board in Southeast Sweden (EPK 757–2021) and the University of Auckland Human Participants Ethics Committee (024414). The researchers had extensive experience in nursing, palliative care, and qualitative research. Their working relationships or prior contact with the participants in the study helped them to develop a rapport during the interviews or focus groups. However, it is important to acknowledge that participants might have attempted to report their experiences more positively as playing an appropriate social role, whereby they believed the researchers wanted to hear [27]. But the diversity and richness of the data, suggests the participants generally welcomed the opportunity to speak their minds. The research team maintained integrity, patient privacy, anonymity and confidentiality of data throughout the study.

## Findings

A total of 12 nurse educators participated, including five from the UK, five from New Zealand and two from Sweden. Participant characteristics are presented in the Table 1 below.

**Table 1:** Participant characteristics

Characteristics	Participants (*n* = 12)
Age range	
35–44 years old	3
45–54 years old	5
55–64 years old	4
Gender	
Male	2
Female	10
Ethnicity	
Swedish	2
New Zealanders of European origin (White)	5
White British	3
Other White	2
Years of work experience in higher education	
1–5 years	1
6–10 years	4
11–15 years	2
16–20 years	3
21–25 years	1
26–30 years	1

Four main themes were generated: (1) impact of visual storytelling, (2) arts-based learning in palliative care, (3) refinement of the graphic novel, and (4) recommendations about future graphic novels.

### Theme 1. Impact of visual storytelling

All participants agreed that a graphic novel is a powerful tool of storytelling within a nursing curriculum. Some participants shared that the story of Vivian is relatable at a personal level, reminding them of their family and their patients.

One participant shared:

“… *the things that I pulled from it would be partly from my own personal experience and partly from being a nurse*,* … those are the things that you’ve got as your background that you’re bringing to it. So*,* you’re looking for*,* or you see*,* so the lack of written detail would actually bring in more discussion is probably what I’m trying to say in a roundabout kind of*.” (N_01).

Another participant added her personal experience:

*“I just want to draw back to the opportunity within a graphic novel to touch on quite a few different things and connect with different people in different ways. Depending on where they’re coming from*,* what their experience is.”* (N_02).

All participants were fascinated with how the speech and thought bubbles depicted conflicting realities.

“… *you need to understand that there are bigger things happening in this person’s life that they may not voice to you. And that might influence their engagement with the service*,* or not*,* or asking for help if it’s needed*.” (N_05).

*“I love the thought bubbles versus actually what she said. And*,* that is*,* the reality of people do filter or are careful around what they will say or think …”* (S_02).

One participant shared the thought about innovative ways of teaching students,

“*I’m trying to think of innovative ways to teach students about fundamental care*,* fundamentals of care*,* and do some kind of tutorials. And wouldn’t it be nice to have something similar to that where a patient is going through an experience of care*,* the healthcare system. And asking students what fundamental care they are receiving and what they are not receiving”.* (N_05)

According to some participants, the graphic novel could also be useful as an inclusive medium for teaching, giving an example of people with learning disabilities,

“… *focusing around people with autism … a real person story*,* you know their voice. And they were saying actually when the nurse (is) asking about their pain*,* they couldn’t express their pain in numbers*,* they could express through colours but that wasn’t an option. So actually something like that … would be really much more inclusive*,* in that approach*.” (N_02).

### Theme 2. Arts-based learning in palliative care

Some participants reflected on how well the novel depicts palliative care and end of life care, where not only Vivian is taking care of her ill husband but also how the physical and mental stress she is experiencing is gradually silencing her. Again, thought bubbles play a role here. Vivian thinks about certain factors she is not able to express that point to such issues as ageism and palliative care.

One participant stressed how the graphic novel - *Vivian* could be used to facilitate discussion and thereby encourage students to think and speak for themselves,

“*I think this is a really powerful way of communicating quite complex issues*,* and stopping yourself from over-reaching as an educator and allowing other people to have their own ideas. So anything that encourages me to allow my students to speak is a good option”.* (S_02)

However, palliative care was not the only discipline in which *Vivian* would be a good fit according to the participants,

“*It could also fit into the specialist training/education for nurses in elderly care (care for older adults). When you study home health care*,* but also*,* at the emergency ward. How do we act? How do we talk to the patients? You wish for another chapter in Vivian’s story. What happens when she comes to the nursing home?”* (T_02).

Another participant shared her thoughts about gender disparity within the field of healthcare and nursing. Gender is an important factor in palliative care, affecting how people access, experience, and receive care. Gender-sensitive care is needed to ensure that everyone receives equitable care, which is one of the key messages to convey through the graphic novel - *Vivian*.

*“… I’ve talked about doing reviews of the curriculum and teaching resources and things like that because of that gender*,* not just gender though*,* to be fair*,* that’s just gender and ethnicity and other characteristics. So*,* I like to see it anyway*,* yeah*,* so I found it (the graphic novel) really interesting*,* the whole reason where it came from and why it’s there*,* it (the graphic novel) was*,* like*,* really good to read.”* (N_02).

Another participant shared similar thoughts and reflections on gender and palliative care – emphasis of the graphic novel,

“*Because I wanted to kind of understand where it had come from*,* and the purpose of it*,* the underlying meaning. I actually read the novel first and then I went back and read it*,* and then read the novel again*,* cause I thought*,* actually then after you read that first part you do kind of have a different spin on it. Particularly focusing on the issue around women*,* older women around palliative care*,* about that caregiving role but also*,* yeah*,* what happens to them when they don’t have a caregiver”.* (N_01)

Furthermore, another participant indicated that Vivian’s experiences, including her treatment at her GP’s office foregrounded behaviour and attitudes of health care professionals towards patients, and attitudes of women towards themselves,

“*I think it is very contemporary oriented. The time in the novel is very much how it is now. That no one has time. Do you have children? Yes*,* I do. But they live far away*,* and she doesn’t want to bother them. And no close friends*,* no acquaintance. It was the neighbour who called the ambulance*,* wasn’t it? And she didn’t know her either. It is a shrinking social network somehow. She keeps a façade. It shines through. The care. She doesn’t get the care she needs. They just belittle her symptoms. She comes to the health centre*,* and no one takes her seriously. You are only allowed to raise one problem*,* and you only have ten minutes. That is what they said to start with*,* she already knew that … she had to pick the problem she thought was the worst. So*,* these problems with a back pain she couldn’t say anything about. She chose the urinary problems.”* (T_01).

Participants who read the graphic novel more than once mentioned their discovery of new insights, which in turn led to stimulating discussions within the group,

“*I do get the power of this. I mean*,* I’m sitting looking at the pictures again*,* and seeing something new each time. So you get something immediate from it*,* discussing it gives you something else*,* looking at the pictures further gives you something else. There’s a lot of thoughtful complexity about the way that the images have been constructed and the decisions that have been made in terms of colour and texture and those kinds of things. So I’d be immensely proud if I’ve been able to produce something like this”.* (S_01)

### Theme 3. Refinement of the graphic novel

According to most of the participants, the graphic novel would be useful if introduced into the curriculum with some revisions to make it more accessible to students. For example, one participant noted *Vivian’s* male and female gender roles seemed stereotyped and thought that the story was really more about ageing than palliative care.

*“I think it’s interesting. I wasn’t convinced this was the ‘bad gender’ as much as age when I was reading it*,* and I would be very interested to see a male-dominated take on the graphic novel because I don’t understand the male perspective as well as I understand the female perspective … It appeared to me to be related to the age of the person and the culture and their background*,* and what their expectations of their role were.”.* (S_01)

Similar views were put forward by another participant,

“*I mean there’s certainly things about her age that has expectations about her role as a woman*,* but I’m not sure. I’m not sure if she was younger*,* we would have those. And also*,* if he was being portrayed*,* he probably [would] have similar stereotyped expectations about his role*,* and so on. And we do know*,* lots of men are the primary carers for women*,* (at) end of life. So yeah*,* I didn’t get the gender thing as much as I got the age thing I’d agree with that”.* (S_04)

A male participant offered a perspective of stereotypes related to gender and men,

*“I feel that a man can experience similar*,* potentially*,* feelings like the lady in this vignette. And so I would argue that men are just suffering in silence*,* and they don’t want any support. But again*,* it might be a stereotype … how we depict male and female roles in here is caring…”* (S_05).

Further refinements might centre on more particularity in regard to each country’s setting in the context of palliative and end of life care,

*“… It’s the kind of that you can’t cope on your own so we’re gonna give you some care. And then the next last picture is oh*,* here we are the retirement village*,* and from a UK perspective that’s really not what happens. And you know*,* really*,* not that quickly*,* but again*,* that’s obviously from a UK perspective.”* (S_02).

Some participants shared that the introduction gave away the surprise element of the story since it contained information about gender inequality and palliative care right at the beginning and therefore the reader knew what to expect. Therefore, participants suggested the reordering of this part of the novel to the end; allowing readers to form their own opinion on what the novel is about.

### Theme 4. Recommendations about future graphic novels

Participants noted that graphic novels could be useful for explaining complex topics such as mental health, communication with patients and relatives, education for nurses in older adult care, home health care, pain management and children’s education programmes.

Mental health in palliative care could be an important topic to centre on in a graphic novel due to its complexity, as this participant notes about Vivian’s experience,

“… *I did wonder*,* cause she’s gone through this journey with her husband and that caregiving. So*,* physically tired but possibly … and probably mentally tired and strained around that. And whether there was any support available for her at that point or even anybody had asked her. And it kind of came pretty clear at the end of the story when she’s talking about not wanting to be a burden on people. And I felt that she just kind of was closing down into a little shell and whether her loss and grief had been explored and she had a chance to talk with anybody about that*,* if that’s what she wanted to do. But*,* yeah*,* maybe support around her mental health and wellbeing*.” (N_04).

Different cultural backgrounds and issues within them could also be considered for future recommendations,

*“I think we need to see more diversity? Some of the nurses could be (from) different backgrounds. Or doctors you know*.” (N_03).

Instead of a textbook like chapter, one participant suggested a slightly different approach to the novel,

“… *like in the textbook where they had the whole chapter and then there’d be a box for questions for reflection or things to do … cause you want them to come to their own conclusions as well. But*,* just to bring in that knowledge that people might not have that helps you think about it a bit more*”. (N_03)

## Discussion

This qualitative study provided nurse educators’ views on how a graphic novel can be used as an interesting medium for nursing education in palliative care, including on issues related to gender. Caregiving is often viewed in society as a woman’s job, however, when the woman needs care, it is often compromised and ignored [28]. There is often gender disparity observed with respect to needs and challenges faced by patients and families receiving palliative care, which may negatively impact on the care they receive [29, 30]. Nurse educators who participated in this study reported that the graphic novel - *Vivian* has expressed these inequalities and issues constructively, and that it is an effective way of teaching undergraduate nursing students about such sensitive topics as palliative care, death and dying.

Education and training on palliative care among health professionals is inadequate [31], and needs to be emphasised in nursing education. Introducing a graphic novel - *Vivian* could help nursing students understand more about palliative care and the complex issues associated. The innovative approaches including the use of graphic novels and digital technology have been developed and used widely in higher education to support teaching and learning [32–34]. As mentioned by Dahlstrom et al., “Educators and institutions need to balance strategic innovation-technology-with solid pedagogical practices and to know students well enough to understand which innovations students value most” ([32], p.34). These findings resonate with nurse educators in the current study who also have highlighted the importance of the use of a graphic novel in an electronic format as a tool for nursing students. Similar findings were reported by Epstein & Bertram where smartphones usage involving technical and adaptive challenges were useful in their curriculum [33]. Kim et al.’s study found positive influence on the skills, knowledge and confidence among nursing students when they had smartphone mobile learning [35].

Use of a graphic novel could be highly influential. Educators suggested that *Vivian*, with its depiction of real-life situations, could support students’ critical thinking with respect to palliative care, gender inequity and other issues. The format of visual storytelling could also be a way to inspire students whose first language is not the same language spoken in the nursing school, or students who have learning disabilities/difficulties [7], as it might be easier to adopt for educational purposes. The opportunities for educators to discuss the graphic novel with students from different levels of learning abilities and understanding [7], can correlate with the theme - Impact of visual storytelling. Similar findings were put forth by Short and Reeves [9] where authors argued that a graphic novel is an important medium which helps students learn effectively because it improves their cognitive ability. O’Flynn Magee and colleagues conducted a study on addressing bullying with the help of a graphic novel among nursing students and concluded that even if creating the graphic novel was a lengthy process, the goal to address bullying was successfully achieved [36]. The social and cultural issues within the society and health care systems were explored by these researchers using a graphic novel as a tool of teaching thus strengthening the advantages of using graphic novels in education. Future research is needed to adapt and refine the graphic novel by considering social and cultural contexts and evaluate how the graphic novel is implemented in nurse education across different settings and countries.

### Strengths and limitations

Strength of this study is that it was conducted in three different countries and common themes were identified and agreed across all sites following regular consultations among researchers. Data were initially coded and analysed by an early career researcher (AD) and cross checked by more experienced researchers (AG and PG). Then themes generated across all the datasets were discussed and agreed with the other project team members. This makes the data transferable in other similar settings.

The use of purposive sampling meant that a diverse range of participants by age, gender, ethnicity and years of experience at higher education were sourced, however, a limitation of this study was that our participants were predominantly from a White population. A more ethnically diverse participant group would provide a broader perspective on *Vivian*’s usefulness as a teaching tool for students.

A strength was also to allow the participants to take part in the study in different ways that were most suitable and convenient for them (e.g., in-person, online, individual interviews, and focus groups). This flexible approach not only maximised participation opportunities, but it also allowed us to explore both shared perspectives in focus groups and nuanced individual experiences in interviews simultaneously. Conducting focus groups only could cause potential risks of limiting the depth of insights and having power imbalance and biased responses. However, adding individual interviews has provided complementary data to focus groups and a more complete picture of the topic [37]. Through the use of focus groups and in-depth individual interviews, this study acts as a good example of data source triangulation in qualitative inquiry [38].

Another strength was the project team worked closely together to ensure credibility, transferability, dependability, and confirmability of the findings. This meant that throughout the analysis a robust critique of the data was collaboratively reflected upon.

## Conclusions

There is scope to use arts-based learning when engaging audiences from diverse backgrounds. The nurse educators stressed that the graphic novel - *Vivian* could help students think ‘outside of the box’. It could stimulate in-depth group discussions as the images used are subject to various interpretations and encourage students to think critically about nursing in palliative care and other healthcare disciplines. Our participants also stressed the importance of an innovative teaching and learning approach that extended beyond textbooks to include other interactive forms, especially those that appeal to Gen Z students (who are considered ‘digital natives’). Using graphic novels in the undergraduate nursing curriculum will help students better understand certain concepts in healthcare, particularly sensitive topics like palliative care. The design of the graphic novel could be refined based on the suggestions by the participants before it can be introduced and evaluated more widely in different languages across the globe. Useful recommendations about different topics (e.g., mental health aspects) could inform the future development of graphic novels.

## Supplementary Information

Supplementary Material 1

## Data Availability

All data generated or analysed during this study are included in this published article. The transcripts can be provided upon reasonable request.
