# Supplementary material for: Evaluation of using a graphic novel Vivian in nursing curriculum from the perspectives of nurse educators: a three-country qualitative study
Source: BMC Palliat Care. 2025 Oct 24;24:270. doi: 10.1186/s12904-025-01907-y (PMC12551296; doi:10.1186/s12904-025-01907-y)
Supplement: Supplementary file 1 — Supplementary Material 1 [file 12904_2025_1907_MOESM1_ESM.docx]

**Interview guide**

**Introduction:**

Thank you so much for agreeing to be interviewed today. My name is ___________ and I am a member of the team working on this study. The purpose of this study is to evaluate the use of a graphic novel *Vivian* in nursing curriculum. Your views will provide important insight into the role, importance, and future development of graphic novels for nurse education and beyond.

The interview should take about an hour. The information you share in this interview will be anonymised, so please feel free to share as much as you would like to. We may cover some difficult/challenging topics during the interview. You can stop the interview at any point or skip any questions should you wish to. Also, we can pause for breaks if you need to, and can restart recording when you are ready.

Could I please just confirm that you are still happy to take part in this interview? And are you happy for the interview to be audio recorded today? And is it ok for me to make a few notes during the interview?

Do you have any questions before we start?

**Discussion:**

1. What do you think this graphic novel is about?
2. What do you think the authors were trying to convey about gender and palliative care?
3. Did you read the introduction? (Why or why not)
4. What (if anything) do you think worked well in the story?
5. What (if anything) could be improved?
6. On what device did you read the graphic novel (e.g., Did they download it to their phone, laptop, desktop computer?) Did reading it on this device work well for you?
7. Do you read graphic novels? If so, what kind (titles, genre) and what devices do you read them on? (phone, e-book, print book, etc.)
8. Do you think graphic novels are a useful way to convey information to nursing students and other healthcare professionals?
9. Would you like to read more graphic novels related to issues that are important to nursing students and other healthcare professionals? (Are they a useful educational tool?)

**Closing:**

Is there anything else you would like to share or add?

I’d like to thank you again for taking the time to be interviewed today, we really appreciate it, and your views will be a great help to us.
